# Barriers to implementing asthma self-management in Malaysian primary care: qualitative study exploring the perspectives of healthcare professionals

**DOI:** 10.1038/s41533-021-00250-y

**Published:** 2021-07-07

**Authors:** Ping Yein Lee, Ai Theng Cheong, Sazlina Shariff Ghazali, Hani Salim, Jasmine Wong, Norita Hussein, Rizawati Ramli, Hilary Pinnock, Su May Liew, Nik Sherina Hanafi, Ahmad Ihsan Abu Bakar, Azainorsuzila Mohd Ahad, Yong Kek Pang, Karuthan Chinna, Ee Ming Khoo

**Affiliations:** 1 UM eHealth Unit, Faculty of Medicine, University of Malaya, Kuala Lumpur, Malaysia; 2 Department of Family Medicine, Faculty of Medicine and Health Sciences, Universiti Putra Malaysia, Serdang, Selangor Malaysia; 3 NIHR Global Health Research Unit on Respiratory Health (RESPIRE), Usher Institute, The University of Edinburgh, Edinburgh, UK; 4 Department of Primary Care Medicine, Faculty of Medicine, University of Malaya, Kuala Lumpur, Malaysia; 5 Klinik Kesihatan Lukut, Ministry of Health Malaysia, Port Dickson, Negeri Sembilan Malaysia; 6 Department of Medicine, Faculty of Medicine, University of Malaya, Kuala Lumpur, Malaysia; 7 School of Medicine, Faculty of Health and Medical Sciences, Taylor’s University, Subang Jaya, Selangor Malaysia

**Keywords:** Health care, Asthma

## Abstract

Asthma self-management is a crucial component of asthma management. We sought to explore healthcare professionals’ (HCPs’) perceptions on barriers to asthma self-management implementation in primary care. We recruited 26 HCPs from six public primary care clinics in a semi-urban district of Malaysia in 2019. The analysis was done inductively. HCPs described barriers that resonated with the “COM-B” behaviour change framework. Capability-related issues stemmed from a need for specific self-management skills training. Opportunity-related barriers included the need to balance competing tasks and limited, poorly tailored resources. Motivation-related barriers included lack of awareness about self-management benefits, which was not prioritised in consultations with perceived lack of receptiveness from patients. These were compounded by contextual barriers of the healthcare organisation and multilingual society. The approach to implementation of asthma self-management needs to be comprehensive, addressing systemic, professional, and patient barriers and tailored to the local language, health literacy, and societal context.

## Introduction

Supported self-management is a crucial component of long-term asthma management^1,2^ in adults that improves clinical outcomes and reduces healthcare costs^3^. Patient-centred, collaborative care that permits effective patient–practitioner communication improves adherence to treatment and outcomes^4,5^. However, studies have shown that, globally, support for asthma self-management is not embedded in routine practice and only a minority of people with asthma have an action plan^6–8^.

In Malaysia, the prevalence of adult asthma was estimated at 5% with asthma-related deaths responsible for 1.2% of all deaths in the 2006 National Health and Morbidity survey^9^. This survey also reported that 20% of adult asthma patients visited the emergency department for acute exacerbations, 10% were admitted, and 27% reported school/work days’ loss with a mean duration of 6 (4–8) days in the past 12 months^9^. Less than half of adult asthma patients had regular long-term follow-up^9–11^. In addition, studies have reported under-utilisation of controller medications^10,11^, while the use of oral short-acting beta-agonist was common among adults with poor asthma control in Malaysia^10–12^.

A wide range of barriers to implementing supported self-management were described in a recent systematic review—these include, poor patient–professional partnership, lack of patient education and concerns regarding medication safety, insufficient professional training, and negative views regarding asthma self-management, compounded by competing priorities and limited time in consultations^13^. Underpinning many of these barriers are challenges to effective communication^13,14^. All but 1 of the 56 papers included in this review were from high-income healthcare systems, reflecting a gap in the understanding of barriers faced in the socio-cultural context of low- and middle-income countries (LMICs), such as Malaysia. Malaysia’s multicultural and multilingual society may also present different barriers to the implementation and delivery of asthma self-management education in primary care settings. As the country is composed of three major ethnicities, Malay (70%), Chinese (22%), and Indian (7%), as well as several minority ethnicities (1%)^15^, a significant proportion of the population reads, writes, and converses in their respective native tongues and exhibit varying levels of fluency in English and the country’s national language, Malay^16–19^. Additionally, the populations’ predominantly “low” to borderline “sufficient” health literacy^20^ and low general literacy skills among the elderly^21^ may have some influence in the barriers experienced. We therefore aimed to explore healthcare professionals’ (HCPs’) views of the barriers faced in implementing supported self-management for asthma in adults in a primary care setting in Malaysia, taking into consideration the country’s cultural and socioeconomical contexts.

## Results

### Participants

We recruited 26 participants. Six focus group discussions (FGDs; 4–6 HCPs in each group) were conducted between July and August 2019 at the Klang District Health Office (5 family physicians, 5 medical officers, 4 each of assistant medical officers, pharmacists, assistant pharmacists, and nurses). Table 1 outlines the demographic data of the participants. There was only one mixed sex focus group among the pharmacists (one male, three females). Other focus groups were all female (family physicians, medical officers, nurses, assistant pharmacists) or all male (assistant medical officers).

**Table 1:** Demographic data of participants (*N* = 26).

		Number
Age (years)	<30	6
	30–39	12
	40–49	5
	50+	3
Sex	Female	21
	Male	5
Ethnicity	Malay	18
	Chinese	2
	Indian	6
Time in current clinical role (years)	<5	6
	5–9	8
	10–14	3
	15–19	5
	20–24	1
	25+	3
Position	Medical officer	5
	Nurse	4
	Pharmacist	4
	Assistant medical officer	4
	Assistant pharmacist	4
	Family physician	5
Used asthma action plans with patients?	No	10
	Yes	16

**Fig. 1:**
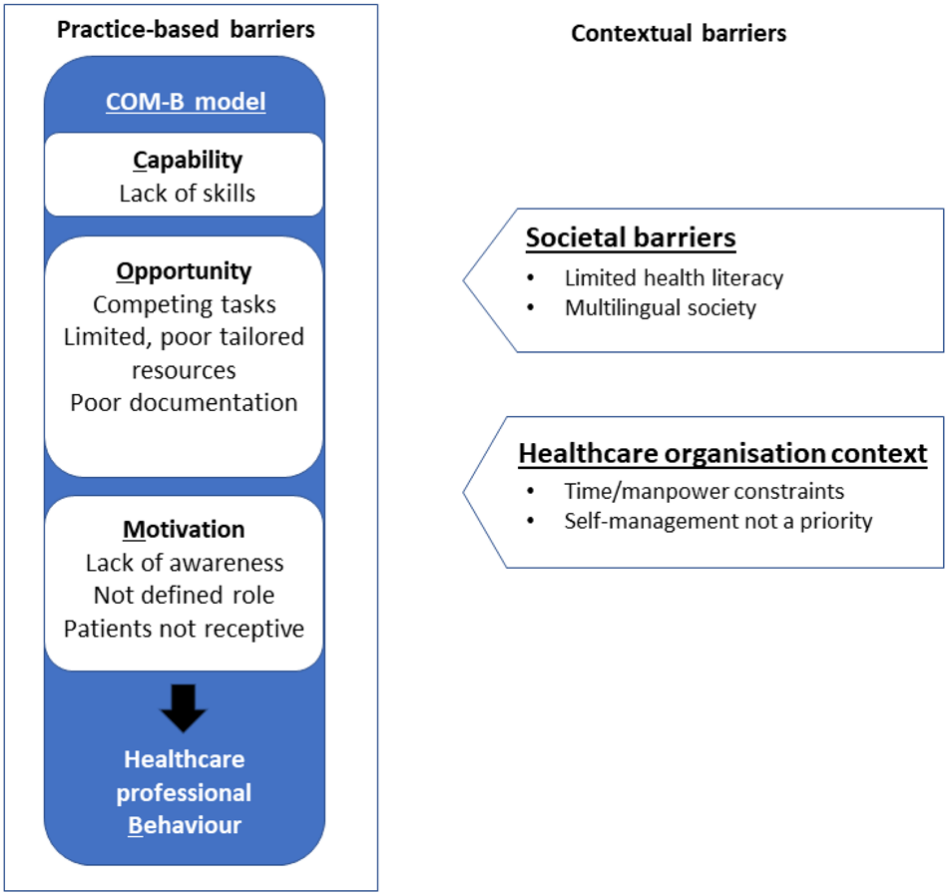
Barriers to implementing asthma self-management education in primary care. Practice-based and contextual barriers to implementing asthma self-management education in primary care.

We identified practice-based and contextual barriers to implementing asthma self-management in primary care practice. Practice-based barriers related to HCPs’ capability, opportunity, and motivation and how these factors influenced their behaviour (COM-B framework). In addition, implementation barriers were influenced by external themes related to societal and healthcare organisational contexts. The interaction of these barriers is illustrated in Fig. 1.

### Practice-based barriers experienced by HCPs

HCPs described a number of barriers to provision of supported self-management that resonated with the COM-B model of behaviour change^22^. Capability barriers included the need for specific skill-based training. Opportunity-related barriers described difficulty in completing tasks due to complex workloads, limited availability, and often poorly tailored resources (e.g. action plans) and poor documentation of previous consultations. Motivational barriers included a lack of awareness of the benefits of supported self-management, hesitancy to stray from their defined roles in the care of people with asthma, and perceptions that patients are not receptive to counselling.

For the barrier on capability, the need for specific skill-based training is one of the important barriers highlighted. The participants acknowledged that without training they would not have the necessary skills to support asthma self-management. Comprehensive training was not provided for all HCPs involved in the management of asthma, which led to knowledge gaps and training inconsistencies among HCPs. Conversely, some participants felt that, while the routine training provided was adequate, the motivation to incorporate into practice was lacking (see below).

“We need more hands-on teaching [of asthma self-management counselling]. Sometimes even the medical officers don’t know how to use the MDI (Metered Dose Inhaler). Some pharmacists don’t know [how to use MDIs] as well.”—A1, Medical Officer, 30–35 years old, 6 years of experience.

“Only a few of our nurses are trained in asthma [self-management counselling]. If they go on maternity leave, and they don’t teach their colleagues… I feel that maybe a class or a course on asthma [self-management counselling] that is detailed and more frequent is necessary to train more nurses.”—A5, Medical Officer, 35–40 years old, 13 years of experience.

In terms of opportunity, competing tasks of the HCPs is another barrier they faced. The HCPs acknowledged that their heavy workload and the multi-condition nature of primary care limited their ability to educate patients on their asthma. The absence of protected time for asthma patient education classes meant fewer opportunities for HCPs to provide self-management counselling as HCPs had to balance their time seeing a very broad range of other patients, as well as addressing other medical conditions in people with asthma.

“We didn’t have any specific dates for asthma [patient education classes], so we have to educate the asthma patients in addition to seeing other patients … [So, the time] to advise patients [is] limited.”—A6, Medical Officer, 30–35 years old, 8 years of experience.

Second, another barrier identified by HCPs related to opportunity was limited availability of resources (asthma action plans) in consultation rooms. This is related to contextual organisational barriers at the district health office level due to budget constraints and lack of prioritisation, which affected the availability of asthma action plans.

“Sometimes, when we want to print out the asthma action plan, we have run out of paper, and we do not have enough money to buy more paper.”—A1, Medical Officer, 30–35 years old, 6 years of experience.

“They will photocopy only a few asthma action plans, only 20 every time. If it runs out, that is it.”—A3, Medical Officer, 25–30 years old, 4 years of experience.

Third, barrier for opportunity also include poorly tailored resources (asthma action plans) available for the patients. The HCPs felt that currently available written asthma action plans were unsuitable for their Malaysian patients as they were too wordy for them to understand, especially considering the high rates of limited health literacy in the population^20^. The HCPs also attributed this dislike towards reading to be a prominent Asian cultural trait. Some suggested that a more visual format of asthma action plans (e.g. pictures or animation) may help patients to understand better.

“The [patient’s] understanding of the asthma action plan is the problem because [it has] a lot of wording here and there.”—F1, Family Physician, 50–55 years old, 26 years of experience.

“Some of them [prefer] visual [formats] to aid them. [If it’s] too wordy [it] is also quite difficult because [in] our culture, they don’t want to… don’t like to read, right?”—F7, Family Physician, 45–50 years old, 21 years of experience.

Lastly, another barrier related to opportunity also includes poor documentation. Some HCPs stated that due to poor medical record documentation it was unclear whether counselling of asthma action plan had previously been provided.

“…also, the documentation is very poor, so we don’t know whether [asthma action plan counselling] is done or not by our MOs, that’s the thing.”—F4, Family Physician, 40–45 years old, 16 years of experience.

For barriers related to motivation, lack of awareness and poor attitude is a major barrier. Some HCPs felt that their colleagues were not motivated to empower patients for self-management even if they had been trained in delivery of action plans, which led to poor attitude towards implementing it.

“We send our medical officers for district training (of asthma self-management counselling). For every session of the CME [Continuing Medical Education], we will ask those who attend the course to go back to their clinic and brief other medical officers who did not have the opportunity to attend. That’s why I believe their training is not too lacking. The issue is just whether they have the motivation to practice it.”—F1, Family Physician, 50–55 years old, 26 years of experience.

This is because counselling of asthma self-management may need extra initiative and time spent by the HCPs, an interrelating systemic issue. Reasons for low motivation overlapped with other barriers, such as the limited provision of training. For example, a pharmacist of 25 years standing had “not heard of” asthma self-management. Limited training on asthma self-management not only affected knowledge of current guidelines but also reduced awareness of the potential benefits for people with asthma having an action plan and thus affected the HCPs’ motivation to improve their practice in empowering patients for asthma self-management.

Unclear HCP roles is another barrier towards motivation of the HCPs. Several HCPs described their lack of motivation as a result of the feeling that supporting self-management was not a responsibility under the clearly defined roles of their job position. Instead, they would refer them to a HCP whose role included self-management counselling. This may represent a lack of awareness and motivation at the management level as most job positions within the clinic’s dedicated asthma team did not include contributing to supporting the self-management.

“New [patients] who need counselling will be directed to the pharmacists. I am a… I am an assistant pharmacist. I don’t counsel them.”—E2, Assistant Pharmacist, 50–55 years old, 25 years of experience.

Another important challenge that reduced HCPs’ motivation is perceived patients’ lack of interest in managing their asthma. HCP felt that asthma patients did not prioritise their self-management and were reluctant to document their symptoms in asthma diaries. The HCPs thought that this problem was more common among patients who were of lower education level, citing examples of patients who were illiterate and were not able to write their symptoms in an asthma diary. It was felt that it would be difficult to empower them to self-manage their asthma without clear record-keeping of their asthma symptom and control monitoring.

“For adults, I feel that they do not care. They do not use their action plans. If you ask them whether they have received a copy of it in their last consultation, [they will reply] ‘I do not remember, Doctor. Maybe it’s at home.”—A1, Medical Officer, 30–35 years old, 6 years of experience.

### Contextual barriers experienced by HCPs

HCPs listed a number of wider contextual barriers that limited provision of supported self-management. At a societal level, the high prevalence of limited health literacy combined with a multilingual society resulted in major barriers in understanding the Malay/English action plans. Healthcare organisations struggled with limited manpower and a lack of priority for asthma self-management.

For the societal context, limited health literacy and language barriers were perceived as major barriers for asthma self-management. There was a widespread belief among HCPs that patients with low educational status and limited health literacy could not understand explanations of an asthma action plan and self-management. This was thought to be a particular problem among older patients. Language was an additional barrier to communication for patients who only understood Mandarin or Tamil as most HCPs could only speak English or Malay. One experienced family physician observed that such patients “might not be able to understand whatever is written in the asthma action plan”. Hence, extra effort in repeated counselling and explanation was needed to enhance the patient’s understanding of an asthma action plan.

“[When explaining self-management to patients], not everyone will [follow] our advice… The more highly educated patients will follow our advice [to regularly take controller medication]. The less educated ones [will only take it] when they feel like it.”—D2, Assistant Medical Officer, 25–30 years old, 6 years of experience.

“In order to deliver your knowledge to your patient, for them to actually be able to understand what you are trying to say, it takes effort and many repetitions (repeated sessions).”—C2, Pharmacist, 30–35 years old, 9 years of experience.

In terms of the organisational priorities, the lack of prioritisation at healthcare service level was highlighted in two ways. First, time, budget, and manpower were limited, with many different health programmes to run, and high patient load constraining opportunities for chronic disease management (as opposed to acute management) and even with good team work there was insufficient manpower to support self-management. It was suggested that management level prioritisation was needed to allocate the resources needed to allow them to spend more time with patients who needed counselling to encourage self-management.

“…time is a big constraint, so if they [ministry management] can intervene and have more staff then they [can] really help us greatly in order for us to spend more time with patient who needs it [counselling of asthma self-management].”—C2, Pharmacist, 30–35 years old, 9 years of experience.

“We all don’t have time to clerk each patient one-by-one. Just to fill the first page [of the clerking sheet] is already difficult. So, we [hoped] the nurses would help us, but the nurses say that they also have a lot of work to do.”—A5, Medical Officer, 35–40 years old, 13 years of experience.

Second, a recurring theme that appeared in HCP discussions was a lack of prioritisation of asthma self-management in favour of other conditions and topics. For example, some campaigns (such as “Know Your Drugs”) had been ongoing for a decade without updating their education materials, but it was also noted that the campaign did not include “how to use” asthma inhalers in their materials. The HCPs attributed the lack of priority to limited awareness on asthma and suggested rotating the focus of the campaign regularly to raise awareness and prioritise asthma from time to time.

“There’s a Diabetes [Awareness] Month, but an Asthma [Awareness Month], I feel that I’ve never seen before.”—F5, 41-year-old Family Physician, 17 years of experience.

## Discussion

This study identifies some significant challenges in the implementation of supported asthma self-management in primary care practice in Malaysia. Barriers that hindered implementation in routine practice were multifactorial, encompassing factors related to capability and motivation of the professionals, as well as practical barriers of resource and time that reduced the opportunity in day-to-day practice. A number of contextual factors external to the clinic in which the HCPs practiced were highlighted: the priorities and resources of the healthcare organisation and the multi-ethnic, multilingual societal context. Many of these challenges could be improved or at least modified with a comprehensive approach to an intervention, which will be further elaborated in the subsequent sections. As highlighted by a systematic review^13^, our findings of professionals’ barriers related to the COM-B framework overlapped with similar themes identified in other studies. Lack of training and poor patient–HCP partnerships limited capability; opportunity was reduced because of pressure of time; and lack of awareness regarding guideline recommendations and action plans, and perceived poor patient receptiveness all reduced motivation, which have all been described in other (typically high-income country) settings^13,23–26^. Our study noted that opportunity was further reduced by limited supplies of poorly tailored self-management resources and poor documentation of whether (or not) self-management had previously been discussed. Lack of awareness about self-management benefits and a hesitancy to work beyond their defined role were additional barriers to motivation. Healthcare and societal contextual factors are identified in other studies^13,23–25,27,28^, but the Malaysian context of a multi-ethnic multilingual LMIC compounded some of these barriers. Lack of awareness about asthma self-management at the level of healthcare management and lack of prioritisation of asthma self-management meant that initiatives did not address the heavy workloads and manpower constraints, for example, by enhancing teamwork. An issue identified in this study was the differing views and expectations on the adequacy of training for HCPs in supporting asthma self-management, a situation that is not limited to a developing country like Malaysia^6,13,23^. Furthermore, we noted a marked separation of roles in the current healthcare provider system, where only doctors and pharmacists were given the responsibility of self-management counselling, which promoted silo-working, poor teamwork, and hesitancy of healthcare providers to support self-management as it was outside their job description. Study participants were recruited from attendees of an asthma training workshop who had been selected to attend because of their direct involvement in the care of patients with asthma. Any negativity elicited within this study may be more pronounced in HCPs less involved (and thus less confident) in asthma care. All these are modifiable barriers. Team-based, comprehensive training of HCPs including nurses, assistant medical officers, and assistant pharmacists in performing—or supporting—asthma self-management counselling may not only facilitate asthma self-management^13,29–32^ but also help overcome the resource limitations. Only some HCPs were able to attend the structured training relating to asthma self-management in the hope that they would share their learning with their peers who had stayed back to carry out clinic duties. Dependence on opportunistic peer training may have led to inconsistent skills and knowledge gaps among some groups of HCPs. Among our study population, none of the four assistant pharmacists (10–25 years of experience) had heard asthma self-management, highlighting how they were not involved in the asthma team and had received minimal training regarding asthma. Their main roles were to dispense medication and check inhaler technique. Training and extending assistant pharmacist and nurse roles within the team may alleviate HCP capability- and opportunity-related barriers. Better training can improve motivation^33–35^. Blended learning using web-based mobile applications can be considered as an option for training delivery as it provides HCPs with increased accessibility to and interactivity with training material and allows for scheduling flexibility for HCPs with heavy workloads^36^. In addition, the use of motivational interviewing, training of lay educators, and group consultations may help to overcome some of the barriers in the delivery of asthma self-management support and care^37–39^. Low levels of literacy and language barriers were perceived as major barriers preventing patients from understanding asthma self-management. Similarly, studies from other countries have reported poor understanding of asthma in South Asian patients and those with low literacy^40–43^, and language barriers prevented HCPs in educating ethnic minority patients on asthma management^23,44^. In addition, the asthma action plan was perceived to be too wordy and information was not available in all the languages widely used in Malaysia. This, in addition to lack of interpretation services, compromised the effective delivery of supported self-management in a multi-ethnic Asian country. However, the perception that literacy is a barrier to self-management may represent a subconscious justification on the part of the HCPs for not offering self-management advice. The use of pictures or videos and the provision of multilingual resources are potential ways to overcome both language and literacy barriers as perceived by HCPs. As reported in other studies, pictorial format tools that offered information in literacy-sensitive manner have been shown to enhance consultations and facilitate understanding of management plan^45,46^, including in Malaysia^47^, and can improve asthma outcomes^48^. In addition, all material should be checked for reading age and readability. Within this programme of work, we are exploring the perspective of patients and co-developing asthma self-management support materials (including paper and mobile format of pictorial asthma action plan) with users. Our participants highlighted that engaging patients in maintaining asthma diaries (as recommended by Malaysian asthma guidelines)^49^ is challenging. This is not unexpected; patients tend to be motivated to manage their asthma when symptoms cause discomfort, affect their daily activities, or if they believe asthma may cause serious consequences^40,50–52^.

Some HCPs in this study suggested internet or mobile monitoring interventions might support monitoring for those interested and able to use such platforms. An example of such an intervention is as a mobile application with graphic icons representing asthma symptoms as visual aid to log daily symptoms^30,44,53–59^. Globally, however, this may not as effective as hoped, as despite prioritising symptom and peak flow diaries as core components of asthma apps, in reality few patients engage regularly with monitoring tasks^60^. Future studies will need to explore the feasibility of using digital support for self-management for asthma in Malaysia. In addition, interventions on a larger contextual scale to promote awareness and understanding of guideline-recommended care include policy changes that support social movements, such as through online health communities that involve patient and public participation, may be the way forward^61,62^. Furthermore, the literacy and language barriers and the lack of training enabling HCPs to overcome these barriers may mean that some patients may not understand the implications of a diagnosis of asthma. Hence, comprehensive education, culturally appropriate, and tailored to the education and literacy level while taking universal health literacy precautions^63^ about asthma and promotion of self-management is necessary. The factors that contributed to the challenges of implementing asthma self-management in a Malaysian primary care setting were multifactorial, but most are potentially modifiable. Interventions will need to adopt a comprehensive approach tailored to the local healthcare system and address the societal context of multiple languages and limited health literacy. Prioritisation of asthma and supported self-management is needed at policy, management, practice, community, and individual levels to enhance access to training, address flexibility of roles, increase awareness, and explore innovative digital approaches to improve supported self-management for asthma. This study included the views of all groups of HCPs in the management of asthma in Malaysian primary care, and our recruitment strategy during an asthma training event achieved a good response. The qualitative study design enabled an in-depth exploration of the barriers that hindered the delivery of supported self-management for asthma among the HCPs. We did not approach professionals who are not involved in asthma care; this could have limited the perspectives heard though we considered that they would have been unlikely to be aware of the issues we wished to explore. Focus groups were organised flexibly, and most of the professionals we invited participated. However, some participants may have hesitated in expressing individual reservations or concerns in the context of a group. Offering the option of one-to-one interviews might have enabled the recruitment of a few more participants and supported more open discussion. To mitigate this, we grouped HCPs of the same role together and experienced qualitative researchers facilitated the discussions to ensure everyone had a chance to contribute. We may thus have missed some factors, especially from the perspective of patients who were not included as participants of this study. The researchers were all primary care physicians with an academic interest in supported self-management, which will have influenced the data collection and analysis. We remained aware of this and discussed emerging findings with a wider group.

## Methods

### Design

This was a qualitative study using FGDs to explore HCPs’ perspectives on the barriers and challenges of supporting patients to self-manage their asthma in their day-to-day practice.

### Healthcare context

Malaysia operates a dichotomous primary care system (public and private). Public primary care clinics are funded through general tax revenues and each consultation costs RM1 (USD 0.20) inclusive of investigations and medication. The private sector is funded by out-of-pocket payments. The public sector provides 60% of outpatient care^64^. In these clinics, a number of HCPs contribute to the provision of asthma management services. Their roles are summarised in Table 2.

**Table 2:** Healthcare professional roles in the provision of asthma management services within public healthcare clinics.

Professional	Roles
Doctors (family physicians and medical officers)	• Assess status of asthma control
	• Manage long-term care of asthma symptoms
	• Prescribe medication
	• Counsel on self-management
Pharmacists	• Assess medication adherence
	• Counsel patients on inhaler technique
	• Counsel on asthma action plan
Assistant pharmacists	• Dispense medication
Assistant medical officers	• Manage acute exacerbations of asthma
	• Refer to medical officers or family physicians for long-term care
Nurses	• Assist in assessment for acute exacerbation of asthma and follow-up care
	• Refer to medical officers or family physicians

### Study setting

The study was conducted in six urban and semi-urban (three urban and three semi-urban) public primary health care clinics in the district of Klang, Selangor, Malaysia that cater to the LMIC populations of the country. At 22%, Selangor has the highest prevalence of adults with asthma in Malaysia^9^. All the six selected clinics are headed by trained family physicians. Each clinic had 11–26 medical officers, 13–55 nurses, 4–8 assistant medical officers, 6–10 pharmacists, and 3–6 assistant pharmacists. This wide variation was related to the size of the clinics and the number of patients attending per day in the clinics; each doctor could expect to see 50–70 patients daily. As part of routine practice, all patients with asthma on follow-up were given asthma dairies to record their symptoms and encouraged to bring the diaries along during their follow-up.

### Participants, recruitment, and sampling

Participants were approached during a workshop on asthma management attended by all the HCPs (30) who were involved in the care of adult patients with asthma in the 6 primary care clinics in the Klang district. Those who agreed to be contacted about the study provided telephone numbers, which were used (via telephone calls or text messages) to confirm their interest to participate and to arrange the focus groups. Composition of the focus groups was according to the participants’ profession to facilitate interaction and avoid hierarchical barriers.

### Data collection

We developed a semi-structured interview guide based on our reading of literature (mostly from high-income countries)^13,14^, our knowledge of the Malaysian socio-cultural context^16–21^, and our experiences in the public health system. The topics covered asthma management and the HCP’s perspectives on the barriers of providing supported counselling on self-management for people with asthma.

We used open-ended questions in the FGDs, with prompts used when important issues did not emerge spontaneously during the interview [Supplementary Information]. Sessions, which lasted between 60 and 90 min, were conducted by P.Y.L., A.T.C., or S.S.G., with field notes on non-verbal cues and interview dynamics taken by an assistant. All interviews were audio-recorded, transcribed verbatim, and checked. Interviews and analyses were performed iteratively until no new themes emerged. Recruitment was stopped after six FGDs when researchers agreed that the analysis had reached thematic saturation.

### Data analysis and validation

We used NVivo 12 software to manage the data. Thematic analysis was used^65^. Data from FGDs and field notes were coded for themes and analysed inductively to identify recurring themes. Comparison of themes both across and within subgroups allowed the understanding of the issues specific to each group. Two researchers (P.Y.L. and A.T.C.) coded one transcript independently and created a list of free nodes (themes). Subsequently, the themes were merged to form categories. The coding was then compared for inter-rater consistency and discrepancies. Any disagreements were resolved through consensus. The final framework was then used to code subsequent transcripts. Any new themes that emerged were added to the list with consensus of the research team. The quotes that best represent the essence of the themes were extracted for inclusion in the “Results” in this article.

### Interpretation and reflexivity

The three researchers (P.Y.L,. A.T.C., S.S.G.) involved in data collection and analysis were female academic primary care physicians. They had frequent open discussions enabling them to reflect on themes and remained mindful of their professional views and biases about asthma self-management support throughout the analysis.

### Ethics approval

This study received ethical approval from the Medical Research and Ethics Committee of the Ministry of Health, Malaysia (NMRR ID: NMRR-18-2683-43494) and sponsorship approval from the Academic and Clinical Central Office for Research & Development (ACCORD) at the University of Edinburgh. All participants provided written informed consent.

### Reporting summary

Further information on research design is available in the Nature Research Reporting Summary linked to this article.

## Supplementary information

Supplementary Information

## Data Availability

The data are not publicly available due to them containing information that could compromise research participant privacy.
