# Supplementary material for: Barriers to implementing asthma self-management in Malaysian primary care: qualitative study exploring the perspectives of healthcare professionals
Source: NPJ Prim Care Respir Med. 2021 Jul 7;31:38. doi: 10.1038/s41533-021-00250-y (PMC8263608; doi:10.1038/s41533-021-00250-y)
Supplement: Supplementary file 1 — Supplementary Information [file 41533_2021_250_MOESM1_ESM.pdf]

Topic guide

Preamble:

- Ice-breaking and explain the aim of the study
- Explain that there is no right or wrong answer
- Explain need to get consent for the interview and audio-recording

Question	Prompts
**Asthma care**	
1. How do you manage asthma in your clinic?	• What do you assess for the control?<br>• Do you know any guideline?
2. How do you decide on the treatment plan for the patients?	• Pharmacological<br>• Non-pharmacological
3. What do you think about the service provided by your clinic to the asthma patients?	• Facilities, human resource (asthma team), medications
**Asthma self-management**	
1. Have you heard about asthma self-management?	• If yes, how do you do that in your practice?
2. What do you think about asthma self-management?	
3. Do you use asthma self-management plan with your patients?	• When do you use? To whom? What types of self-management plan? In what format (eg. paper, apps, ICT) and which format do you prefer?<br>• If no, why? How can this be improved?
4. Do you have problems in using asthma self-management plan with your patients?	• If yes, as healthcare providers what are the problems using the plan? Do your patients have problems using the plan? How can this be improved?<br>• If no problem, what facilitate you in using it? Do you know what facilitate your patients in using the self-management plan?<br>• What format that you prefer? Website/mobile apps/social media?<br>• How does ICT help?<br>• Would that be applicable in your practice?
5. Is there any role of information and communication technology (ICT) in the delivery of asthma self-management plan?	• If yes, could you explain further how would you approach them differently?<br>• What about the patients that you decide to approach them differently? (e.g. different approach uses for different age

6. Does your asthma care differ from one patient to another?	group, ethnicity, socioeconomic status, and literacy level)
